# OrthoDB v8: update of the hierarchical catalog of orthologs and the underlying free software

**DOI:** 10.1093/nar/gku1220

**Published:** 2014-11-26

**Authors:** Evgenia V. Kriventseva, Fredrik Tegenfeldt, Tom J. Petty, Robert M. Waterhouse, Felipe A. Simão, Igor A. Pozdnyakov, Panagiotis Ioannidis, Evgeny M. Zdobnov

**Affiliations:** 1 Department of Genetic Medicine and Development, University of Geneva Medical School, rue Michel-Servet 1, 1211 Geneva, Switzerland; 2 Swiss Institute of Bioinformatics, rue Michel-Servet 1, 1211 Geneva, Switzerland

## Abstract

Orthology, refining the concept of homology, is the cornerstone of evolutionary comparative studies. With the ever-increasing availability of genomic data, inference of orthology has become instrumental for generating hypotheses about gene functions crucial to many studies. This update of the OrthoDB hierarchical catalog of orthologs (http://www.orthodb.org) covers 3027 complete genomes, including the most comprehensive set of 87 arthropods, 61 vertebrates, 227 fungi and 2627 bacteria (sampling the most complete and representative genomes from over 11,000 available). In addition to the most extensive integration of functional annotations from UniProt, InterPro, GO, OMIM, model organism phenotypes and COG functional categories, OrthoDB uniquely provides evolutionary annotations including rates of ortholog sequence divergence, copy-number profiles, sibling groups and gene architectures. We re-designed the entirety of the OrthoDB website from the underlying technology to the user interface, enabling the user to specify species of interest and to select the relevant orthology level by the NCBI taxonomy. The text searches allow use of complex logic with various identifiers of genes, proteins, domains, ontologies or annotation keywords and phrases. Gene copy-number profiles can also be queried. This release comes with the freely available underlying ortholog clustering pipeline (http://www.orthodb.org/software).

## INTRODUCTION

Orthology is the cornerstone of comparative genomics and gene function prediction. The availability of gene sequence data from a large variety of species is growing quickly, and the gap between such sequence data and the experimental functional data is widening. The evolutionary relatedness of genes, termed homology, can be asserted by sequence analysis, providing the means to formulate working hypotheses on gene functions from experimentation on model organisms. In turn, homologs referencing a particular ancestor have been termed orthologs (1–3). Such genes originating by speciation from an ancestral gene are most likely to retain the ancestral function (4), making orthology the most precise way to link gene functional knowledge to a much wider genomics space. Assessment of gene orthology is also instrumental for interpretation of whole-genome shotgun metagenomics (5) that is reshaping microbiology with a direct impact on future medicine (6).

The term ‘orthology’ was initially coined for a pair of species having just one common ancestor (1). Expanding this concept to a group of species (2–3,7), OrthoDB aims to identify groups of orthologous genes that descended from a single gene of the last common ancestor (LCA) of all the species considered. Such generalization includes not only genes descended by speciation from the LCA, but also all their subsequent duplications after the radiation from the LCA, i.e. co-orthologs. Applying this concept to the hierarchy of LCAs along the species phylogeny results in multiple ‘levels of orthology’ with varying granularity of orthologous groups. While it is possible to obtain more finely resolved orthologous relations for some pairs of species that radiated after the clade's LCA (i.e. referring to a younger LCA), generalization over more than two species brings greater power for integrating sparse experimental functional data.

The central role of the orthology concept prompted the development of numerous approaches and resources (8). Due to the challenges of inferring orthology and scalability of the methods, however, there are few resources (9,10) that match OrthoDB in scope (Table 1) and only a small set of available orthology delineation software (discussed below), prompting the wide use of an oversimplified approach (11) that selects only one out of possibly multiple co-orthologs.

**Table 1.** Organism coverage of the major resources providing orthology

	Number of genomes				
Database	Total	Bacteria	Eukaryotes	Orthology levels	Availability
OrthoDB.v8	3028	2627	401	270	GUI, data, software
eggNOG.v4	3686	2031^a^	238	107	GUI, data
KEGG-OC	3098	2675	256	n.a.	GUI

^a^Used to define orthologous groups.

**Figure 1.**
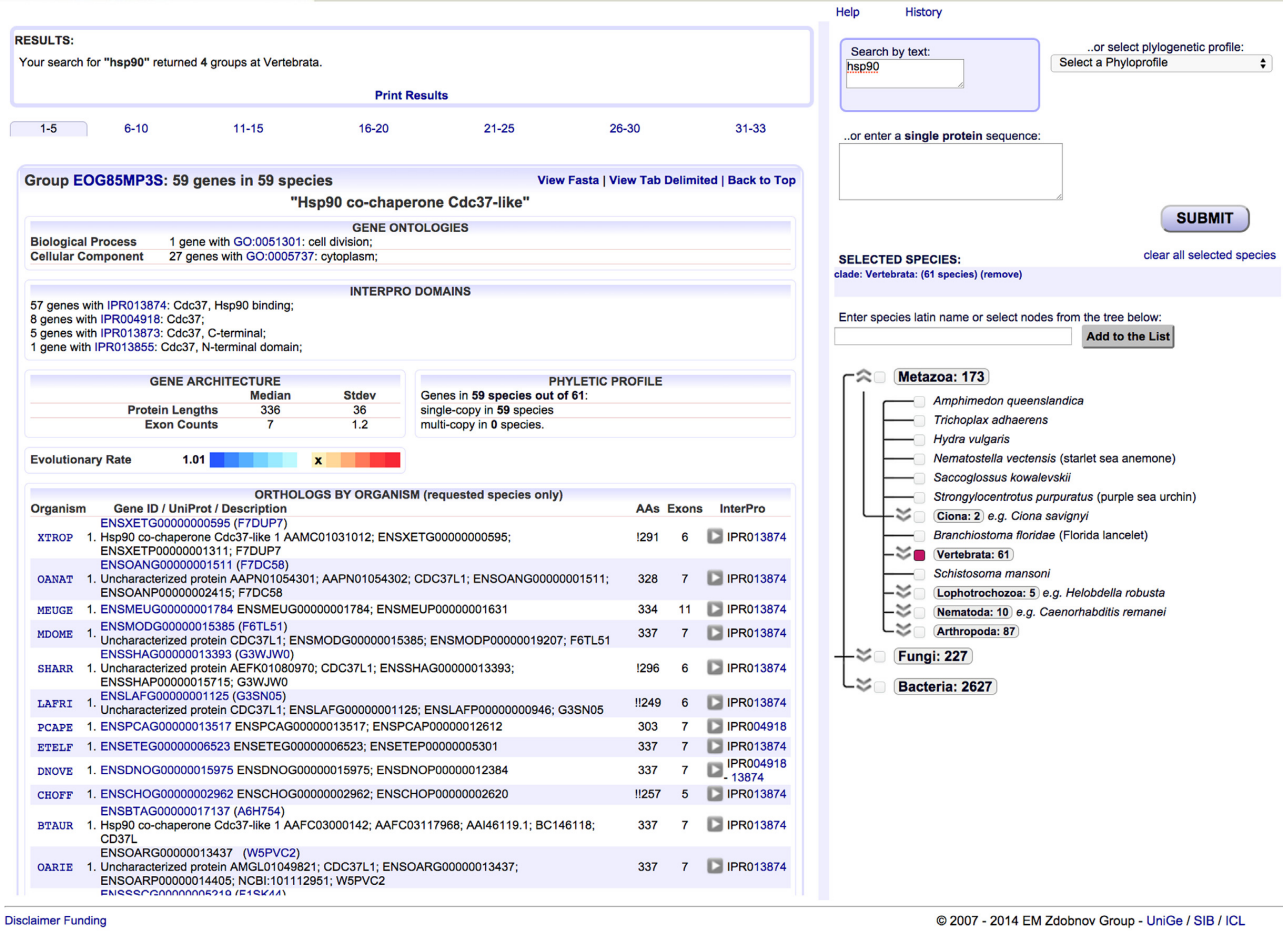
OrthoDB web user interface. The orthologous group centric results panel is on the left and the query-building panel is on the right.

OrthoDB is one of the largest resources of orthologs in terms of number of genomes covered, and has promoted the concept of hierarchical orthology since its conception (12). In this release we re-implemented the OrthoDB website and the graphical user interface (GUI) (Figure 1). Similar to some other resources, OrthoDB provides tentative functional annotations of orthologous groups and mapping to functional categories. Notably, OrthoDB provides the most extensive collection of functional annotations of the underlying genes linked to their original sources. Gene annotation is a complicated process that is hardly feasible without automation, which in turn can introduce errors. Although in many cases OrthoDB makes such errors in the collated annotation data apparent, search results with particularly discordant annotations should be considered with caution. The evolutionary annotations of the orthologs and statistics of gene architectures remain the distinguishing features of OrthoDB.

## COVERAGE OF EUKARYOTIC AND PROKARYOTIC GENOMES

The current update brings OrthoDB to the same level as the leading orthology resources (outlined in Table 1), covering 2627 bacterial, 227 fungal, 61 vertebrate, 25 basal metazoan genomes and the most comprehensive set of 87 arthropod genomes. Of the total of almost 10 million bacterial and over 5 million eukaryotic protein-coding genes analysed, 91% and 89% of them respectively were classified into orthologous groups. Evolutionary annotations were computed for all groups, where about 80% of the groups have functional annotations sourced from specialized resources. Since orthology is relative to the LCA, we identify orthologous groups at the major radiations within each lineage comprising 28 animal, 40 fungal and 202 bacterial levels of orthology. OrthoDB now uses the NCBI taxonomy (13) to define levels of orthology.

Protein-coding gene annotations for vertebrates were retrieved from Ensembl (14) (Release 75, February 2014). Arthropod data were retrieved from AgripestBase, AphidBase (15), BeetleBase (16), DiamondBackMoth-DB (17), Ensembl Metazoa (18), FlyBase (19), Hymenoptera Genome Database (20), NCBI (13), SilkDB (21), VectorBase (22), wFleaBase (23), as well as the i5k pilot project (24) and several other genome consortia (July 2014). Gene sets for the additional basal metazoan species were retrieved from Ensembl Metazoa (18) and the Joint Genome Institute (25) (July 2014). The fungal gene sets were sourced from UniProt (26), (February 2014 release). We retrieved over 11,000 bacterial genomes from Ensembl Bacteria (Release 22, May 2014), and selected 2627 with the most complete annotations and the best sampling of the genetic diversity using a set of universal single-copy genes and our BUSCOs pipeline (Simao *et. al*., submitted).

## THE ALGORITHM AND SOFTWARE

With this update we provide the suite of programs for delineation of orthologous genes that was developed for, and is the basis of, the OrthoDB hierarchical catalog of orthologs. The suite includes an efficient clustering procedure scalable to thousands of genomes as well as a multi-step pipeline to handle the complete data analysis flow. The package is distributed under the BSD License from http://www.orthodb.org/software.

OrthoDB ortholog delineation is a multi-step procedure. First, best reciprocal hits (BRH) of genes between genomes are identified (which represent the shortest path through the speciation node between these genes on a distance-based gene tree). Second, matches within each genome that are more similar than the best reciprocal matches between genomes are identified (these represent gene duplications after this speciation point, i.e. co-orthologs). The third and final step involves triangulating and clustering all BRHs and in-paralogs into groups of orthologous genes. Such clusters, called orthologous groups, represent all descendants of a presumably single-gene of the LCA of all the species considered. As in previous releases, this update considers only the longest isoform per gene. Technically, the OrthoDB software suite contains two packages: (i) a collection of Bash and Python scripts that implement the multi-step data analysis pipeline and (ii) an efficient rule-based clustering of the BRHs into groups of orthologous genes written in C++. The data analysis pipeline with pluggable external software currently employs SWIPE (27), implementing full Smith–Waterman pair-wise sequence alignment algorithm, and CD-HIT (28) for identification of very similar gene copies.

## BENCHMARKING

Not many methods (29–31) are available for gene orthology delineation that can scale to hundreds of genomes (Table 2, Supplementary Table S1). Usually, there is a trade-off between the precision (‘getting only the right ones’) and recall (‘getting all the right ones’), and different objectives may favour a particular compromise. For example, having insufficient precision may result in propagation of erroneous annotation or diminishing phylogenetic signal, while insufficient recall will give only a fragmented view inappropriate for comparative genomics studies as well as diminished possibilities for annotation propagation. Moreover, since orthology is defined evolutionarily and the true gene and species histories are not known, there is no clear baseline for comparison of alternative orthology predictions. One approach to benchmark alternative orthology predictors is to compare the results against a human-curated classification (32). We refer to this reference classification below as RefOGs. Although such curated orthologs comprise only a small subset of organisms and gene families (prompting discussions to what extent such a subset of challenging cases is representative of all gene families in complete genomes) and can include uncertain expert decisions, this benchmarking approach remains the most appropriate option in our view. There are four other alternatives. The first is to compare concordance of predicted orthologs with available functional annotations. However, this only describes the evolution of gene's functions rather than the evolution of genes themselves, and this measure can be variable among gene families and functions. The second is to compare concordance of gene genomic arrangements (of slowly shuffled genomes, e.g. mammals, or of gene arrangements under selection, e.g. operons in bacteria). Yet this only provides evidence of orthology, not of non-orthology. The third approach is to compare concordance with InterPro domains, or the Gene Ontology (GO) annotations frequently inferred from them, which only provide evidence of incorrect orthologous group fusions (e.g. by erroneously fused gene model predictions) since more broadly defined homologs are compared to more narrowly defined homologs (arisen only after a particular LCA). The fourth approach is to compare concordance among different methods, which is biased by technical similarities. A common benchmarking fault is to compare orthologs predicted for different sets of organisms referring to different LCAs; these are inherently different by definition. An example would be to compare orthologous groups to pair-wise orthology that does not span the most ancient radiation in the group.

**Table 2.** Comparative performance of available orthology calling methods versus RefOGs (32)

					RefOGs		
Method	Num. of OGs (RefOGs=67)	RefOGs with F1 ≥85%	RefOGs with Presicion ≥85%	RefOGs with Recall ≥85%	Sum: Exact, Akin	Sum: Fused(events), Split(events)	Sum: Complex, Missed
OrthoDB v8 (2014)	112	51	67	46	43: 30, 13	45: 0(0), 20(45)	4: 4, 0
OrthoDB v5* (2010)	156	42	67	34	33: 24, 9	89: 0(0), 30(89)	4: 4, 0
OrthoMCL (2.0.8)	124	45	64	49	40: 30, 10	51: 2(1), 20(58)	5: 4, 1
COGsoft (4.2.3)	164	29	66	19	19: 12, 7	64: 0(0), 28(64)	20: 19, 1
OMA (0.99t)	224	20	66	13	12: 8, 4	134: 0(0), 31(134)	24: 23, 1

* Used in prior benchmarking (32).

F1 is a harmonic mean of precision and recall (http://en.wikipedia.org/wiki/Sensitivity_and_specificity). RefOG events are defined as follows: ‘Exact’–having 100% of both precision and recall; ‘Akin’–having precision and recall >85% (i.e. up to 1 ‘wrong’ gene for 37% of RefOGs and up to 2 ‘wrong’ genes for another 20% of RefOGs); ‘Fused’–counting fusing events when more than one RefOG represented one method cluster with RefOG recall >85% and summed method cluster precision >85%; ‘Split’–defined symmetrically to Fused when one RefOG is represented by more than one method cluster; ‘Complex’–when the matches can not be classified into another category; ‘Missed’–when a RefOG recall <50%.

The most direct comparison to RefOGs is to apply methods to only the data that was used for curating the RefOGs (i.e. the same sequences as seen by the curator) and then compare the obtained grouping to RefOGs. The comparison of alternative clustering (grouping) can be considered in a few respects: (i) as the number of special cases of group-level fusions and splits considering only matches with high precision that are less undesirable than complex matches or misses (Table 2), (ii) as the fraction of RefOGs that matched better than a certain degree of precision or recall (Table 2) and (iii) as pair-wise metrics of overall concordance between alternative clustering methods (Table 3). These results are surprisingly consistent with less direct comparison of predictions made on complete current gene sets and the mapped RefOGs to the current data (Supplementary Table S2), even though only 93% of RefOG sequences could be unambiguously mapped to current gene annotations.

**Table 3.** Concordance on ‘Variation of Information’ between the methods and RefOGs (lower values indicate more similar classifications)

	Reference	OrthoDB.v8	OrthoDB.v5	OrthoMCL	COGsoft	OMA
Reference	0	7.7	12.5	10.3	17.3	20.6
OrthoDB.v8	7.7	0	6	7.7	12.1	15.4
OrthoDB.v5	12.5	6	0	7.5	9.1	11.5
OrthoMCL	10.3	7.7	7.5	0	9.9	13.9
COGsoft	17.3	12.1	0	9.9	0	10.4
OMA	20.6	15.4	9.9	13.9	10.4	0

## FUNCTIONAL AND EVOLUTIONARY ANNOTATIONS

*Functional annotations* are arguably the most sought-after information. The extent and detail of available functional gene annotations varies considerably and are mostly only available for genes in model organisms. OrthoDB has provided such annotations for each gene since the first release, avoiding any automated propagation of potentially spurious annotations. The practical utility of annotations at the level of orthologous groups is hard to define, however, and thus we provide some automatic annotations with a disclaimer that they should be treated as explicitly tentative without an expert validation.

*SUCCINCT FUNCTIONAL DESCRIPTORS* of orthologous groups are derived by summarizing frequently occurring annotation terms or phrases mapped to individual member genes.

*FUNCTIONAL COG CATEGORIES* were assigned to each orthologous group, whenever possible, by mapping of the GO terms (33) to manually curated COG (7) functional categories (from http://geneontology.org/external2go/cog2go). Such high-level functional descriptors are informative for creating concise functional profiles for comparative genomic and metagenomic studies (GO slim and subset guide; http://geneontology.org/page/go-slim-and-subset-guide).

*GO TERMS AND INTERPRO DOMAINS* are summarized over the member gene annotations. GO terms (33) for molecular function, biological process and cellular component were mapped from UniProt (26) and InterPro (34) protein domain signatures were sourced from the UniProt Archive (UniParc) and computed for new eukaryotic species that are not yet in UniParc.

*DOMAIN ARCHITECTURES* are presented as sequentially ordered InterPro domains from the N- to C-terminus for each member gene. This enables searches for specific domain combinations as well as facilitates visual inspection of the conservation of protein domain architectures across all members of the orthologous group.

*GENE SYNONYMS AND PHENOTYPES* are highlighted, whenever available, in the results table of orthologs with direct links to their respective source databases (Figure 1). The data were retrieved for selected model species from each of the major lineages: *Caenorhabditis elegans* from WormBase (35), *Danio rerio* from the Zebrafish Model Organism Database (36), *Drosophila melanogaster* from FlyBase (19), *Mus musculus* from the Mouse Genome Database (37) and *Saccharomyces cerevisiae* from the *Saccharomyces* Genome Database (38).

*HUMAN DISEASES* associated with particular genes from the online Mendelian inheritance in man (OMIM^®^) (39) resource are also mapped and linked to the original records.

*ESSENTIAL GENES* from 16 bacteria were retrieved from the Database of Essential Genes (40) and include *Escherichia coli*, *Haemophilus influenza* and *Mycobacterium tuberculosis*, and additionally from EcoGene (41) for *E. coli* genes.

*Evolutionary annotations* are computed from available genomics data. Gene families evolve under varying levels of constraint on their sequence identity and gene copy-number (42) that is presumably indicative of their rates of possible changes in functional load, and consequently the confidence of extrapolating hypotheses of gene function from experimentally studied genes. For example, functional inferences are more confident for conservative orthologous groups that show near-universal single-copy distributions and relatively low sequence divergence than for dynamic orthologous groups with patchy phylogenetic distribution, or numerous duplications, or high sequence divergence. OrthoDB provides quantifications of the following evolutionary traits:

*PHYLETIC PROFILE reflecting universality and duplicability.* Universality refers to the ortholog phyletic profile, i.e. genes present in all, most or only a few species. Duplicability refers to retention of gene duplicates that independently happened in all, most, few or no species. The profiles (Figure 1) therefore quantify the maintenance of orthologs across the phylogeny as well as their propensity for gene duplication throughout the evolutionary history since their LCA.

*EVOLUTIONARY RATE reflecting constraints on protein sequence identity.* Quantification of relative sequence conservation among orthologous genes is computed by averaging over all inter-species protein sequence identities normalized by the average identity of all BRHs for each species pair. This correlates with other evolutionary traits, e.g. sequence evolution of single-copy orthologs is more constrained than that of multi-copy orthologs, and with functional traits, e.g. orthologous groups with essential genes usually exhibit more conservative sequence evolution than those without (42).

*GENE ARCHITECTURE reflecting observed variations of protein lengths and exon counts.* Summary of median and standard deviation values for protein lengths (in all lineages) and exon counts (in metazoa) for all genes in each orthologous group provide ‘canonical’ gene architectures of each group. Comparing protein lengths and exon counts of each member gene to the canonical architecture can highlight deviations indicative of inaccurate gene models or dynamic intron evolution.

*SIBLING GROUPS reflecting the sequence uniqueness of the orthologs.* Orthologous groups presumably represent the gene content of a particular ancestral lineage, some of which may have originated by earlier gene duplications (i.e. paralogous genes) or they can share only a duplicated fragment, e.g. evolutionarily mobile domain. Such homology relations among ‘sibling’ orthologous groups are noted in this OrthoDB release by shared content of InterPro domains, requiring at least two organisms from each group to have a shared domain. Orthologous groups with no or very few siblings are unique or rare in the gene universe, while those with many siblings belong to large gene superfamilies where orthology delineation can be the most challenging.

## THE WEB INTERFACE

With this release we re-implemented the OrthoDB web interface to be sustainable with data growth. The main organi*z*ation has remained similar, with the query-building panel now on the right and orthologous group centric results panel on the left (Figure 1). The query-building panel allows:

*TEXT SEARCHES* by protein, gene, InterPro, GO identifiers, UniProt accession numbers, etc., as well as names, synonyms and functional terms or phrases (quoted). Gene annotations were sourced from UniProt and supplemented with data from specific resources for representative model organisms. The text search also allows the use of logical operator syntax to build complex queries; e.g. to optionally include variations of a term, or to exclude terms. In addition, specific protein domain architectures may be queried with a comma-separated N- to C-terminus ordered list of InterPro identifiers.

*COPY-NUMBER PROFILE SEARCHES* by predefined lists of specific gene copy-number phyletic profiles, such as ‘all single-copy’ or ‘all multi-copy’ orthologs.

*SEQUENCE SEARCHES* by BLAST homology search of user provided protein sequence in FASTA format against gene sequences used to build OrthoDB. If significant matches are found, the corresponding orthologous group closest to the root-level is returned.

*ORTHOLOGY HIERARCHY LEVEL*, in addition to the search options outlined above, can be specified by the user by entering/searching for species of interest or by checking/unchecking radiation nodes of the depicted NCBI species classification. As noted above, orthology is relative to a particular LCA. Therefore, results will contain broader groups of genes when ancient radiations (nodes closer to the root) are selected, and narrower gene correspondences for more closely related species. To enable the most precise comparative studies, OrthoDB has always promoted this concept of hierarchical orthologous groups by computing orthology at different phylogeny radiations.

The results panel (left) is orthologous group centric, i.e. if OrthoDB is queried using a gene identifier, the orthologous group containing this gene is returned as the result. Each orthologous group has a unique identifier (in the style of EOG8xxx and POG8xxx in this v8 release). First, when available, the collated functional annotations are displayed, including InterPro and GO terms, followed by the computed evolutionary annotations outlined above. This is followed by a list of the corresponding orthologous genes with their original annotations. The results can be printed or viewed as tab-delimited text, and the gene sequences can be viewed in FASTA format.

## DATA ACCESS

As for the previous versions of OrthoDB, in addition to the web interface we provide data files for bulk download, one file per level of orthology; as well as the underlying gene sequences in FASTA format, and mapping of the genes to UniProt, NCBI and RefOGs. All data are distributed under the Creative Commons Attribution 3.0 License from http://www.orthodb.org/. Users can also navigate to OrthoDB records by following links from FlyBase ‘Orthologs’ section, UniProt ‘Phylogenomic databases’ section or NCBI ‘Additional links/ Gene LinkOut’ section.

## CONCLUSIONS AND PERSPECTIVES

The rapidly growing number of sequenced genomes increases the power of comparative analyses, but also brings new challenges for the scalability of methods and the data presentation to end-users. The most significant highlights of this update are: (i) the technical revamp of the OrthoDB web interface, (ii) the introduced selection of the input data by its completeness and uniqueness, where less complete genomes or accumulating well-represented transcriptomes will only be mapped to the previously defined orthologous groups and (iii) release of the underlying orthology delineation software to enable the research community to perform more specific/private projects or to analyse organisms not yet covered. OthoDB will continue, to the best of available resources, to provide comprehensive coverage of publicly available genomes and to refine the accuracy of ortholog delineations.

## SUPPLEMENTARY DATA

Supplementary Data are available at NAR Online.
